# Complement propagates visual system pathology following traumatic brain injury

**DOI:** 10.21203/rs.3.rs-3970621/v1

**Published:** 2024-02-23

**Authors:** Davis Borucki, Baerbel Rohrer, Stephen Tomlinson

**Affiliations:** Medical University of South Carolina; Medical University of South Carolina; Medical University of South Carolina

## Abstract

**Background:**

Traumatic brain injury (TBI) is associated with the development of visual system disorders. Visual deficits can present with delay and worsen over time, and may be associated with an ongoing neuroinflammatory response that is known to occur after TBI. Complement activation is strongly associated with the neuroinflammatory response after TBI, but whether it contributes to vision loss after TBI is unexplored.

**Methods:**

Acute and chronic neuroinflammatory changes within the dorsal lateral geniculate nucleus (dLGN) and retina were investigated subsequent to murine controlled unilateral cortical impact. Neuroinflammatory and histopathological data were interpreted in the context of behavioral and visual function data. To investigate the role of complement, cohorts were treated after TBI with the complement inhibitor, CR2-Crry.

**Results:**

At 3 days after TBI, complement C3 was deposited on retinogeniculate synapses in the dLGN both ipsilateral and contralateral to the lesion, which was reduced in CR2-Crry treated animals. This was associated with microglia morphological changes in both the ipsilateral and contralateral dLGN, with a more amoeboid phenotype in vehicle compared to CR2-Crry treated animals. Microglia in vehicle treated animals also had a greater internalized VGlut2+ synaptic volume after TBI compared to CR2-Crry treated animals. Microglia morphological changes seen acutely persisted for at least 49 days after injury. Complement inhibition also reduced microglial synaptic internalization in the contralateral dLGN and increased the association between VGLUT2 and PSD95 puncta, indicating preservation of intact synapses. Unexpectedly, there were no changes in the thickness of the inner retina, retinal nerve fiber layer or retinal ganglion layer. Pathologies were accompanied by reduced visual acuity at subacute and chronic time points after TBI, with improvement seen in CR2-Crry treated animals.

**Conclusion:**

TBI induces complement activation within the dLGN and promotes microglial activation and synaptic internalization. Complement inhibition after TBI in a clinically relevant paradigm reduces complement activation, maintains a more surveillance-like microglia phenotype, and preserves synaptic density within the dLGN. Together, the data indicate that complement plays a key role in the development of visual deficits after TBI via complement-dependent microglial phagocytosis of synapses within the dLGN.

## Introduction

Traumatic brain injury (TBI) is a significant cause of long-lasting disability in both children and adults. Visual disturbances are an important sequela of TBI that can severely impact quality of life outcomes (1). Vision problems are reported to occur in as many as 70% of concussion patients (2) and 65% of TBI patients (3), and encompass a wide range of manifestations including visual acuity loss. Vision loss can be immediate and severe, as in cases involving traumatic optic neuropathy (4), or can be more gradual and develop or worsen over time. The latter is due principally to the fact that after the primary injury, there is a secondary injury phase consisting of ischemia, oxidative stress, and inflammation that leads to spreading of injury and further structural and functional decline (5). The ongoing neuroinflammatory response involved in secondary injury after TBI is a potential target for therapies that could prevent further development or worsening of disease over time.

The complement system is a key contributor to secondary injury after TBI. Complement can be activated via three pathways, the classical, lectin, and alternative pathways, which initiate differently but converge at a C3 cleavage step. Our laboratory has previously shown that inhibition of C3 cleavage with the injury site-targeted inhibitor CR2-Crry reduces neuroinflammation in the motor cortex and hippocampus, with corresponding improvement in cognitive deficits at acute and chronic timepoints after TBI (6–9). CR2-Crry is a fusion protein that consists of the extracellular C3-binding domain of complement receptor 2 (CR2), which localizes the construct to sites of complement activation, linked to the murine complement inhibitor Crry, which functions at the central C3 cleavage step and therefore prevents local complement activation without systemically affecting complement activity (10). C3 activation products resulting from C3 cleavage, namely soluble C3a and C3 opsonins, can propagate inflammation. C3a can promote immune cell recruitment and activation via C3a receptor engagement, and membrane-bound C3 opsonins can be recognized by microglial complement receptors leading to recognition and clearance of C3 opsonized cells and material (11, 12). Microglia activation is associated with transition to a more amoeboid morphology, with increased phagocytic capability and with production and secretion of neurotoxic products that further promote inflammation (13, 14). Microglia have been shown to phagocytose C3-opsonized tissue, including synapses, in perilesional regions following brain injury, which correlated with cognitive decline (9). While the complement system has been studied in the perilesional area and ipsilateral hippocampus after TBI, complement activation in the dorsal lateral geniculate nucleus (dLGN) and its potential contribution to visual decline after TBI is unexplored.

Previous work investigating the visual system after TBI has been limited due to the use of animal models that have such severe damage that it resulted in complete functional vision loss, or by study paradigms that lacked any functional vision measures. Also, previous work focused solely on the retina and optic nerve as the sites of pathology, and thalamic or cortical effects were not investigated. In contrast, there are many studies identifying thalamic changes after human TBI, and to this end we investigated whether the dLGN is an important site of inflammation and secondary injury after murine TBI, and further whether this contributes to poor visual outcomes. We also investigated whether C3-dependent microglial-mediated synaptic loss occurred within the dLGN and whether this was linked to post-TBI visual outcomes. The work reported herein was performed in the context of a therapeutically relevant paradigm of complement inhibition.

## Results

### TBI causes vision loss 10 days after injury

**Figure 1:**
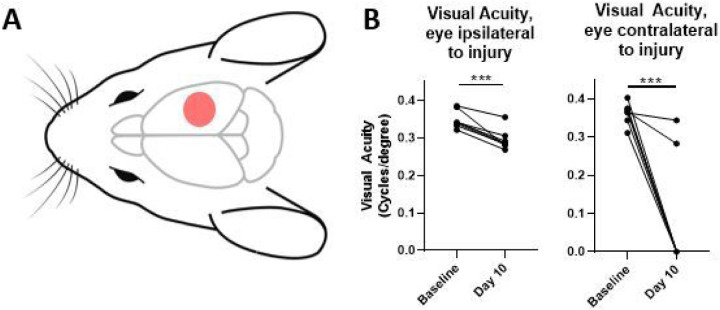
Controlled cortical impact produces a significant decline in visual function. (a) Schematic of injury location. Mice receive a craniotomy and a single, direct, right-sided impact on the dura (B) Mice experience deficits in visual acuity as measured by the optomotor response after injury, with most mice experiencing a complete loss in contralateral vision, and a significant decrease in ipsilateral visual acuity. b, paired t-test. ***p<0.001.

Following unilateral right-sided controlled cortical impact (CCI, Fig. 1A), visual acuity was assessed using a virtual optometry testing system in unrestrained animals that tracks the reflexive head movement in response to a moving grating and allows for spatial acuity testing in the two eyes independently (15). Note that based on the laterally placed eyes of the mouse, almost all retinal ganglion cells cross at the optic chiasm into the contralateral dLGN, and at 10 days after TBI the majority of mice had complete vision loss in the contralateral eye. In contrast, mice developed a partial, but significant reduction in visual acuity in the ipsilateral eye after TBI (Fig. 1B).

### Vision loss is associated with acute inflammation and removal of C3-opsonized synapses by activated microglia at 3 days post injury

**Figure 2:**
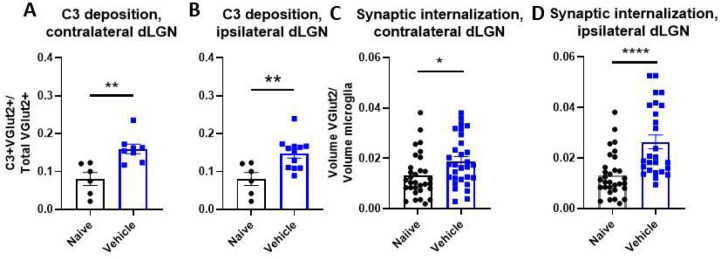
Controlled cortical impact results in increased complement deposition on retinogeniculate synapses and increased synaptic internalization by microglia (a-b) Proportion of synapses colocalizing with C3 in the dLGN contralateral (a) and ipsilateral (b) to injury. (c-d) Microglial internalization of excitatory retinogeniculate synaptic marker VGLUT2 in contralateral (c) and ipsilateral (d) dLGNs. a-d t-test, *p<0.05, **p<0.01. Error bars = mean ± s.e.m.

We have previously demonstrated that activation of the complement system in the perilesional hippocampus is associated with cognitive decline after TBI. To investigate a potential role for complement in vision loss as measured at 10 days after TBI, we examined C3 deposition on retinothalamic synapses at an earlier time-point in both the ipsilateral and contralateral dLGN. These synapses contain the excitatory presynaptic membrane protein vesicular glutamate transporter 2 (VGLUT2). At 3 days after TBI we identified a close association (< 0.2 μm) of C3 + puncta with VGLUT2 + puncta, indicative of C3 deposition on retinogeniculate synapses. The fraction of colocalizing C3 + VGLUT2 puncta was increased in both the contralateral and ipsilateral dLGNs of vehicle treated TBI mice as compared to naïve mice (Fig. 2A,B).

C3 opsonization has been shown to guide microglial phagocytosis within the central nervous system. This C3-dependent process can be beneficial for the clearance of cellular and myelin debris, and it is important for synaptic pruning during normal development. However, aberrant activation of this system occurs following brain injury and can lead to the removal of viable neurons and their synapses (6, 8, 9, 16–19). We assessed microglial internalization of synapses by calculating microglial phagocytic index (i.e., the volume of VGLUT2 + synapses engulfed by microglia) (16). Engulfed VGLUT2 + synaptic material was elevated in both the ipsilateral and contralateral dLGN, (Fig. 2C,D). Taken together, the above data suggest that complement is activated in the dLGN shortly after TBI, resulting in C3 opsonization of visual circuit synapses and their subsequent phagocytic removal.

### Complement inhibition reduces acute inflammation and synapse removal within the dLGN

**Figure 3:**
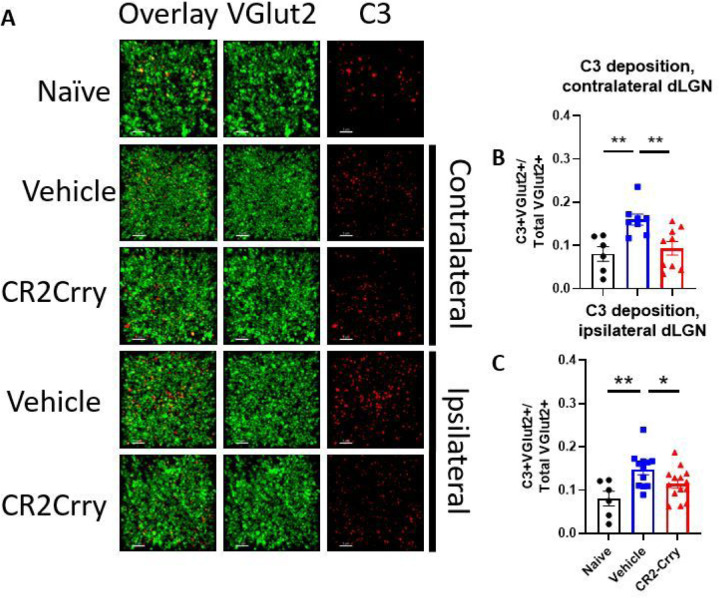
Complement deposits on synapses in the dLGN, both ipsilateral and contralateral to injury, and is reduced by complement inhibition. (a) Representative images showing C3 (red) and VGLUT2 (green) colocalization within the dLGN. Scale bar = 5 μm. (b) Proportion of synapses colocalizing with C3 in the contralateral dLGN. (c) Proportion of synapses colocalizing with C3 in the ipsilateral dLGN. , t-test, *p<0.05, **p<0.01. Error bars = mean ± s.e.m.

**Figure 4:**
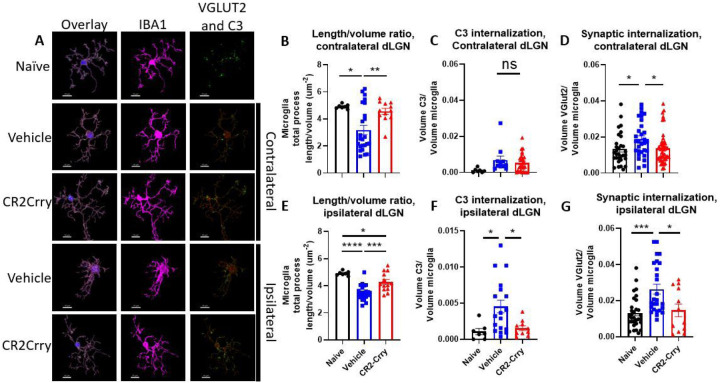
Microglia internalize VGLUT2+ synapses and C3 after injury and display morphological features indicative of activation in both dLGN, and these changes are reduced by complement inhibition. (a) Representative microglial reconstructions with internalized VGLUT2 (green) and C3 (red), and microglial morphology (IBA1, magenta). Scale bar = 10 μm. (b) Microglial filament length to volume ratio, (c) microglial internalization of C3, and (d) internalization of VGLUT2 in the contralateral dLGN. (e) Microglia filament length to volume ratio, (f) microglial internalization of C3, and (g) microglial internalization of VGLUT2 in the ipsilateral dLGN. c, f: t-test. b, d, e, and f: one-way ANOVA with Tukey correction for multiple comparisons. *p<0.05, **p<0.01, ***p<0.001, ****p<0.0001. Error bars = mean ± s.e.m.

Having established that synaptic deposition of C3 occurs within the dLGN acutely after TBI, we investigated whether the subsequent phagocytic removal of C3-opsonized synapses was complement-dependent. To this end, we treated a cohort of animals with the injury-site targeted complement inhibitor, CR2-Crry, at 1 h after TBI. The inhibitor is administered systemically (i.v. injection), but localizes specifically to sites of complement activation, including within the brain after TBI (9, 10). We first demonstrated that CR2-Crry treatment significantly reduced the association of C3 with VGLUT2 puncta relative to vehicle treated controls, in both the ipsilateral and contralateral dLGN (Fig. 3). We next investigated microglial morphology which can be used as an indicator of microglial activation status; homeostatic microglia possess long, dynamic filamentous processes that surveil the surrounding tissue, and upon activation undergo morphological changes that include a retraction of processes and a reduction in branch complexity (13). Following TBI, microglia exhibited a decrease in total filament to volume ratio in both the ipsi- and contralateral dLGN, suggesting filament retraction indicative of microglia activation (Fig. 4A, B). Furthermore, microglia in the ipsilateral dLGN of vehicle treated TBI animals exhibited increased C3 internalization compared to naïve animals, (Fig. 4F), whereas no significant difference in C3 engulfment was detected between the groups in the contralateral dLGN (Fig. 4C). However, engulfed VGLUT2 + synaptic material was elevated in both the ipsilateral and contralateral LGN (Fig. 4D, G), and decreased with complement inhibition. Taken together, these data suggest that following TBI complement is activated within the dLGN, and that there is a complement-dependent process of microglial activation and microglial internalization of C3 opsonized synapses. Further, this process can be inhibited by a single systemic dose of CR2-Crry administered acutely after TBI.

### Complement inhibition preserves visual acuity and cognitive function chronically after TBI

**Figure 5:**
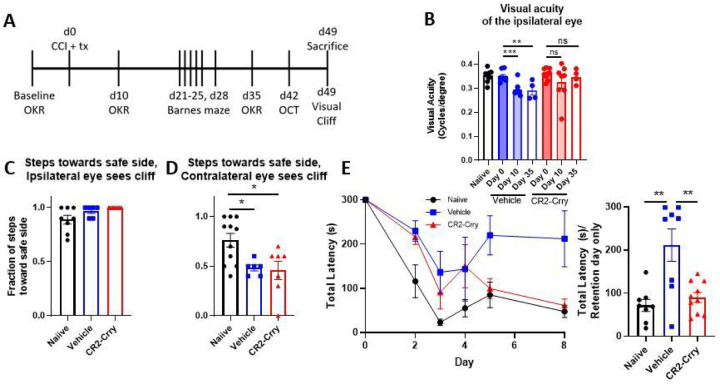
Injury site-targeted complement inhibition prevents the formation of visual deficits in the ipsilateral eye after controlled cortical impact. (a) Treatment paradigm. CR2-Crry (16 mg/kg) or saline was administered via i.v. tail vein injection one hour after CCI. Behavioral testing was performed at the timepoints indicated. (b) Acute inhibition of complement activation preserved visual function in the ipsilateral eye, as measured by the optomotor response. (c) Animals almost always step away from the cliff when it is in their ipsilateral field of vision. (d) Both vehicle and CR2-Crry-treated animals step toward the cliff or safe sides with equal frequency when the cliff is in their contralateral field of vision. (e) Acute inhibition of complement activation significantly reduced time to find the escape hole in the Barnes maze. b-e one-way ANOVA with Tukey correction for multiple comparisons. *p<0.05, **p<0.01, ***p<0.001. Error bars = mean ± s.e.m.

We next investigated whether the acute effects of complement inhibition seen within the dLGN after TBI (reduced complement activation, reduced microglia activation and reduced internalization of C3 opsonized material) translated to long-term improvement in visual function. Mice were subject to TBI and treated with either CR2-Crry or vehicle in the same paradigm as above, and cognitive and vision tests performed over a 7 week period (refer to Fig. 5A for timeline).

Visual function was assessed using the optomotor response described above. In response to a moving stimulus, mice will reflexively turn their head to track the stimulus, integrating information from the movement of the visual image across the retina and only requiring retinal input and integration of the information at the level of the brain stem, but not at the level of the cortex (20). Visual acuity was assessed at both 10 and 35 days after TBI. Vehicle treated TBI mice exhibited a significant reduction in visual acuity in the eye ipsilateral to injury relative to their own baselines, as shown above. However, in CR2-Crry treated animals, visual acuity at both time points after injury was similar to that measured at baseline (Fig. 5B). Near-complete vision loss was observed in the contralateral eye at both time points irrespective of treatment (data not shown).

Visual function was also assessed using the visual cliff test, which also requires visual input in addition to visual cortex activity. In this test, mice are placed on a clear plastic platform, which overlays a safe appearing side and a side that appears to be a cliff. Mice with normal vision are expected to step towards the safe side with higher frequency, whereas mice with worse vision step toward both sides with equal frequency. Given that our TBI mice have different visual phenotypes in each eye, their behavior was also assessed depending on which direction the mouse originally faced. When mice started the task with the ipsilateral (less-affected) eye facing toward the cliff, both vehicle and treated mice consistently went toward the safe side, similar to naïve mice (Fig. 5C). However, when mice started the task with the contralateral eye facing toward the cliff, both vehicle and CR2-Crry treated mice stepped toward both sides at equal frequency (Fig. 5D). This suggests that despite the presence of acuity deficits for finer visual discrimination, large field vision was similar between both groups and not affected by complement inhibition.

For confirmation of brain injury in our TBI model, cognitive ability was assessed using the Barnes maze. The Barnes maze is a spatial memory and learning task in which mice are trained to identify an escape hole over the course of 5 days, and then their spatial memory is assessed 2 days after the final training day. The learning phase of this test depends on reference to large visual cues, which are high contrast and within the visual acuity range of the ipsilateral eye of both vehicle and CR2-Crry animals. The Barnes Maze requires visual input, hippocampal function and frontal cortex function (21). Vehicle treated TBI mice performed significantly worse at this test than naïve or CR2-Crry treated animals, taking significantly longer to reach the escape hole on the retention day (Fig. 5E). As noted, this test depends on visual cues, but the results from the visual acuity (OKR) and visual field data (Visual Cliff) shown above indicate that the worse performance in this task by vehicle vs CR2-Crry treated animals is due to cognitive and not visual deficits.

### Complement inhibition preserves synaptic density in the contralateral dLGN and attenuates microglial morphology changes after TBI

We next determined whether the acute changes observed in microglial morphology and phagocytic activity 3 days after TBI persisted chronically (49 days after TBI), and whether acute complement inhibition affected these chronic outcomes. We show here our analyses of the contralateral dLGN, since there was a complete loss of vision in the eye contralateral to injury and no functional benefit of complement inhibition in the ipsilateral dLGN. Data for the ipsilateral dLGN is shown in supplemental material.

**Figure 6:**
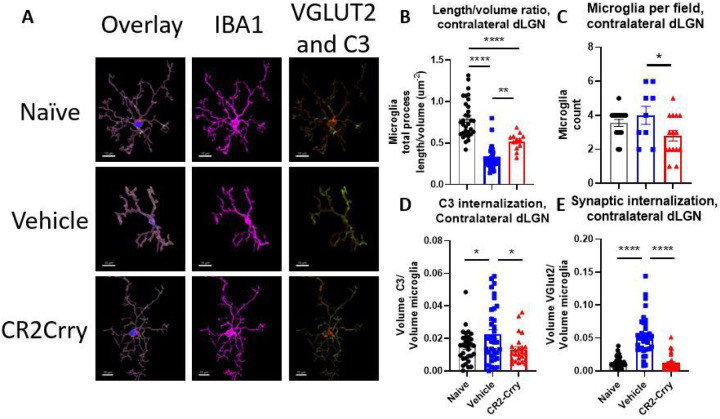
Microglia in the contralateral dLGN still internalize VGLUT2+ synapses and C3 and display morphological features indicative of activation along with increased numbers at a chronic timepoint. (a) Representative microglial reconstructions with internalized VGLUT2 (green) and C3 (red), and microglial morphology (IBA1, magenta). Scale bar = 10 μm. (b) Microglia filament length to volume ratio in the contralateral dLGN. (c) Microglia count per 63x high power field. (d) Microglial internalization of C3 and (d) microglial internalization of VGLUT2 in the contralateral dLGN. B, d-e one-way ANOVA with Tukey correction for multiple comparisons. c t-test. *p<0.05, **p<0.01, ***p<0.001, ****p<0.0001. Error bars = mean ± s.e.m.

At 49 days after TBI, microglia in both the ipsilateral and contralateral dLGN continued to exhibit significant changes in their filament length to volume ratios in vehicle treated mice (Fig. 6B, S1). Microglial morphological changes were partially, but significantly reversed with CR2-Crry treatment, although microglial morphology did not return to parameters measured in naïve mice. There were also significantly more microglia per field in the ipsilateral and contralateral dLGN in the vehicle treated mice compared to naïve or CR2-Crry treated mice (Fig. 6C, S1). Together, these data indicate increased microglial number and dysfunction at a chronic timepoint after TBI. The microglial phagocytic index was calculated to assess whether microglia were still internalizing C3 opsonized or synaptic material at this later timepoint. Microglia in the ipsilateral dLGN of vehicle treated TBI mice showed no significant change in internalized C3 relative to naïve or CR2-Crry treated animals (Fig. S1). However, microglia in the contralateral dLGN of vehicle treated mice showed a significant increase in internalized C3 relative to both naïve and CR2-Crry treated animals (Fig. 6D). Microglia similarly showed increased internalization of VGLUT2 + synaptic material in the contralateral, but not ipsilateral dLGN in vehicle treated TBI mice (Fig. 6E).

**Figure 7:**
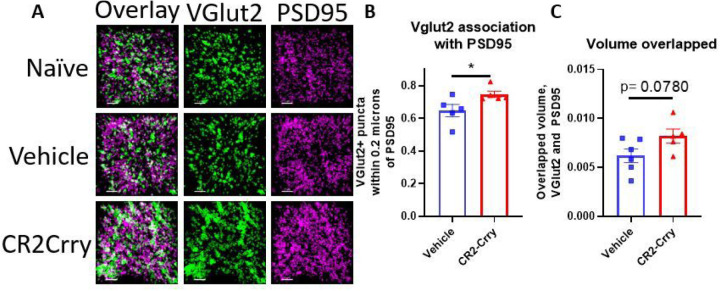
Synaptic density in the contralateral dLGN is preserved by complement inhibition. (a) Representative images showing VGLUT2 (green) and PSD95 (magenta) colocalization within the contralateral dLGN. Scale bar = 5 μm. (b) Proportion of VGLUT2 puncta within 0.2 μm of PSD95 puncta. (c) Total volume overlapped between VGLUT2 and PSD95. t-test, *p<0.05. Error bars = mean ± s.e.m.

Given that complement inhibition reduced microglial internalization of VGLUT2 in the contralateral dLGN at both the acute and chronic timepoints, we wanted to see if the corresponding protection in visual acuity could be explained by differences in synaptic density. Notably, preservation of visual circuit synapses has been associated with visual acuity preservation in demyelinating disease (22).We measured the fraction of VGLUT2 + synaptic puncta within physiologic distance of the post-synaptic marker PSD95, indicative of a structurally intact synapse. There was a significant reduction in VGLUT2 measured in close proximity (< 0.2 μm) to the postsynaptic marker PSD95 (Fig. 7B), as well as a trend towards a preservation in volume overlap of VGLUT2 and PSD95 (Fig. 7C) in vehicle vs. CR2-Crry treated animals. Overall, these data suggest that microglial synaptic internalization is associated with loss of intact retinogeniculate synapses, but that these changes exhibit differences in timing based on localization. In the ipsilateral dLGN, which exhibits complete functional loss, complement-mediated removal of synapses occurs quickly and then levels off, whereas in the contralateral dLGN with less vision loss, synapse removal is prolonged. In the contralateral dLGN, complement inhibition is associated with a reduction in synaptic internalization by microglia at both an acute and chronic timepoint and a preservation of structurally intact synapses, suggesting that long term complement-dependent synaptic remodeling may be contributing to the observed decline in visual acuity.

Supplemental Fig. 1: Inhibition of the complement system reduces microglia counts and partially attenuates morphological changes chronically in the dLGN ipsilateral to injury without affecting synapse or C3 internalization. (a) Representative microglial reconstructions with internalized VGLUT2 (green) and C3 (red), and microglial morphology (IBA1, magenta). Scale bar = 10 μm. (b) Microglial internalization of VGLUT2 and (c) microglial internalization of C3 in the ipsilateral dLGN. (d) Microglia count per 63x high power field. (e) Microglia filament length to volume ratio in the ipsilateral dLGN. b-c, e one-way ANOVA with Tukey correction for multiple comparisons. D t-test. *p < 0.05, **p < 0.01, ***p < 0.001, ****p < 0.0001. Error bars = mean ± s.e.m.

### Effect of TBI and complement on retinal inflammation

**Figure 8:**
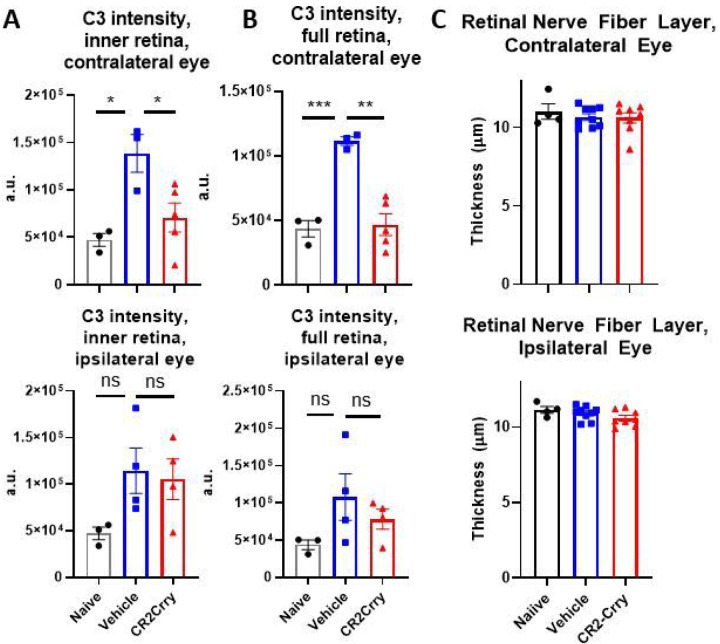
Controlled cortical impact does not cause appreciable changes in retinal thickness, but does result in increased complement staining in the retina contralateral to injury. (a) There is an increase in C3 staining intensity in the contralateral eye, both in the inner retina and full thickness retina, that is reduced by complement inhibition. There are no significant differences detected in the ipsilateral retina. (b) There are no significant changes in the thickness of the retinal nerve fiber layer of either eye at 42 days after injury, as measured by optical coherence tomography in living animals.

Damage to the LGN can cause retrograde retinal ganglion cell degeneration and retinal inflammation. Indeed, retinal inflammation and changes in retinal ganglion cell density have been described in several models of murine TBI (23–26). We examined inflammatory changes in retinas collected from mice 7 weeks after TBI, at which time visual function remained impaired. An increase in C3 staining intensity was detected in both the inner retina and total retina contralateral to the injured hemisphere in vehicle treated mice compared to CR2-Crry treated and naïve mice (Figs. 8A, B). In comparison, no significant changes in C3 intensity were detected in the inner retina or the total retina ipsilateral to the injured hemisphere. In the contralateral eye, the increase in C3 deposition in both compartments of the retina was attenuated in CR2-Crry treated animals, suggesting that some inflammation occurs in the retina after injury and is partially prevented by complement inhibition. We also assessed whether this increase in C3 staining intensity is associated with a neurodegenerative effect. In human patients, a decrease in retinal nerve fiber layer thickness has been shown to occur over time after injury, suggesting that there is ongoing retrograde neurodegeneration in the visual system as a result of TBI (27). We measured the thickness of the retina in living mice using optical coherence tomography 42 days after TBI. We did not observe any differences in thickness of the retinal nerve fiber layer (Fig. 8C), inner retina, or total retina (data not shown), indicating that although there is some inflammation present in the retina, there is no significant degeneration at this timepoint. Together with our data showing both early and late inflammatory changes in the dLGN, our data indicate that the dLGN is the primary site of pathology after TBI, at least in the current model.

## Discussion

Traumatic brain injury is associated with significant disability and mortality. It consists of a primary injury phase that can result in focal tissue damage and diffuse axonal damage, and a secondary inflammatory phase consisting of cellular and biochemical responses to the primary injury that can last for years (28). Axonal damage can affect any neural tract, including the optic radiation (29). Visual impairment is strongly associated with TBI, and one study of military personnel with blast injury reported vision problems in 65% of patients (3), with another study reporting visual acuity changes in 13% of TBI patients (30). Alterations in the visual system can occur long after injury, and retrograde degeneration of retinal ganglion cell axons was detected two months after injury in one case study (31). Another study reported ongoing decline in retinal nerve fiber layer thickness in a longitudinal cohort following patients over several years (27). Visual problems associated with TBI are heterogeneous and can vary depending on the type of injury and its severity (32), but vision impairment represents an important functional outcome that should be included in preclinical and clinical studies.

In this study we used a moderate to severe model of TBI consisting of a right-sided controlled cortical impact to the dura mater. The laterality of the impact results in a differential vision loss phenotype in each eye. In the mouse, 90% of optic nerve axons cross at the optic chiasm, meaning each LGN primarily receives monocular input from the contralateral eye, with a small area of binocular input, integrating information from the ipsilateral eye. In this study, the eye contralateral to the injured hemisphere had the greater vision loss phenotype, with a majority of mice showing no optomotor response to a clockwise-rotating pattern at any spatial frequency. In contrast, the ipsilateral eye retained vision, albeit with impaired spatial acuity. Likewise, when mice were placed in the visual cliff apparatus such that the cliff side was in the field of view of the contralateral eye, they went to either side with roughly equal probability (50:50% chance behavior), again indicating complete vision loss in the contralateral eye. The ipsilateral visual field driven response in the cliff test was not affected by TBI. Vision tests examining performance of the contralateral eye in either the Optomotry or in the visual cliff task were not improved by complement inhibition. This complete contralateral vision loss could be due to primary injury affecting the LGN or parts of the optic nerve/optic radiation, or to a complement-independent component of secondary injury. Optic nerve damage at the site of the optic canal has been described in a weight drop model of TBI, although no functional vision measures were reported (33). The eye ipsilateral to the injured hemisphere had a less severe visual phenotype, with a significant but incomplete decrease in the optomotor response to a counterclockwise-rotating pattern observed at 10 and 35 days after injury. Vision loss in the ipsilateral eye was completely attenuated by complement inhibition, suggesting that complement activation after the primary injury contributes to vision loss in this eye. Notably, in the visual cliff task, all animals, regardless of TBI injury and/or CR2-Crry-treatment, consistently avoided the cliff when initially placed such that they could see the cliff with the ipsilateral eye, suggesting that the decrease in visual acuity in the vehicle group does not affect large object discrimination. This is noteworthy in the context of the Barnes maze test since this is a measure of cognitive performance that relies on spatial memory in reference to large visual cues. Thus, the data taken together indicate that cognitive performance measured by the Barnes maze was not significantly impacted by visual field impairment after TBI, but rather by the level of damage to the hippocampus (34).

There is support for the dLGN being a relevant area of pathology after TBI. In a population of TBI patients with predominantly blast injuries, deficits in functional connectivity between the LGN and other brain regions was observed on functional MRI (35). Also, a blast-TBI study in male Sprague Dawley rats identified microglial and astrocytic activation in several nuclei of the thalamus, although they did not look at the LGN specifically or visual outcomes (36). In the current study, we identified complement activation and signs of microglial activation in the LGN acutely after TBI, both in the injured and contralateral hemispheres. Moreover, deposited C3 colocalized with visual circuit synapses which was accompanied by increased microglial internalization of C3 and synaptic material. This suggests that synapses within the dLGN are being selectively pruned in a complement-dependent manner, including in the hemisphere contralateral to injury. The synaptic marker used here was VGLUT2, a specific marker of excitatory retinal ganglion cell inputs in the LGN. A decrease in the presence of this synaptic marker is associated with decreased visual system function, and it is known to be pruned in a complement-dependent manner during normal post-natal development, as well as in demyelinating disease in which it is also associated with decreased visual acuity (22, 37). In addition to synaptic internalization, we observed a significant decrease in both the fraction of VGLUT2 + synaptic puncta within physiologic distance of PSD95 + puncta in vehicle treated TBI mice, and a decrease in the overall volume of colocalized pre- and post-synaptic puncta in the contralateral dLGN. These data suggest that synaptic changes occur in the dLGN after TBI and persist for weeks after injury, and may be related to the persistently increased microglial presence and internalization of synaptic components in the region. The dynamics of synapse loss and recovery after TBI are complex (34, 38, 39), with an initial loss of synapses in the hippocampus followed by a period of synaptogenesis and recovery of synapse numbers (34). In addition, a loss of excitatory-inhibitory balance is also commonly reported in TBI (39). These observed synaptic density changes in the dLGN may be associated with the complement-dependent decline in visual function observed in this half of the visual system. We showed that microglial morphological alterations were sustained at 7 weeks after TBI, with continued microglial internalization of C3 and synaptic material in the contralateral dLGN. A single acute post-TBI treatment with CR2-Crry reduced these inflammatory and synaptic changes in the dLGN, including chronically after TBI. Although the changes in the ipsilateral LGN did not correspond to a protection of visual function in the contralateral eye, the changes in the contralateral LGN did. Preserved visual acuity in the ipsilateral eye was associated with a reduction in complement deposition and reduced synaptic and C3 internalization in the contralateral LGN.

Despite the relevance of the LGN and thalamus to human post-TBI visual system deterioration, the majority of murine TBI studies have focused on changes in the retina and optic nerve. In a recent systematic review of murine blast-TBI, 35 identified studies investigated changes in the retina and optic nerve, and none examined changes in the LGN (40). A study using a milder closed head weight drop model and following mice up to 5 months after injury identified increased fluoro-Jade staining identifying degenerating neurons and astrogliosis in the dLGN and vLGN at multiple timepoints, although they did not detect differences in the soma area of microglia, and microglia morphological changes or counts were not assessed in detail (41). However, this is a different type of injury and is also significantly milder than the TBI model used in the current study. To note, in our model we found minimal changes in the retina at chronic timepoints after TBI. We did not detect significant changes in the thickness of any layers of the retina in either eye at 6 weeks after injury, including the retinal nerve fiber layer and inner retina. We did detect a slight increase in C3 staining intensity in the contralateral retina that was attenuated in the CR2-Crry-treated animals, but we did not detect significant differences in C3 staining intensity in the ipsilateral retina. Together, these data indicate that the LGN is an important site of post-TBI pathology and neuroinflammation that is responsible for vision impairment, and that the dLGN deserves increased attention in animal models of TBI examining defects in the visual system.

There are limitations to the current study. This study included only male mice. Of note, differences in the cellular makeup of the inflammatory response and complement expression has been noted in murine spinal cord injury, with male mice having a higher microglia to macrophage ratio and increased C1qa expression (42). A study specifically investigating sex differences in murine CCI identified a stronger inflammatory response in male mice up to 7 days post-injury (43), although contributions of the complement system were not investigated. These studies suggest that the inflammatory nature of CNS injury and contributions of the complement system may vary with sex. The current study also focused primarily on changes within the dLGN. Traumatic brain injury induces diffuse inflammation throughout the brain, and potentially in different areas of the visual system, such as the hippocampus, visual cortex or retina (we did examine the latter). Our administered complement inhibitor does not specifically localize to the LGN, but rather any site where there is ongoing complement activation (C3 deposition). Therefore, although we detected reduced complement activation, attenuated inflammation, and microglial changes in the LGN, it is possible that complement activation/inhibition in other parts of the visual system may contribute to the measured visual outcomes.

In conclusion, the complement system is an important contributor to the secondary injury phase of TBI. Our lab has previously shown that complement activation promotes neurodegeneration and cognitive decline after TBI, and that C3 activation represents a potential therapeutic target for treating TBI (6, 8, 9). In the current study, we show that C3 inhibition also improves visual acuity outcomes after TBI, and that that the dLGN represents an important target of C3 inhibition for improving vision. We show that after TBI there is increased complement deposition, changes in microglial morphology, and microglial complement and synaptic internalization within the dLGN. Taken together with our previous data analyzing microglial activity within TBI lesion penumbra (REF), our findings suggest that aberrant synaptic phagocytosis occurring within the dLGN may contribute to vision loss, which can be reversed with complement C3 inhibition. There are currently no therapeutic interventions for TBI, and the only visual interventions available involve optomotor rehabilitation, tinted or prismatic lenses, or behavioral adaptations (32). There is currently a C1-inhibitor being investigated in a clinical trial for TBI (CIAO@TBI), and although it does not specifically include visual outcomes in its primary or secondary endpoints, it includes quality of life score, which may partially reflect improved visual outcomes (44). Note that while C1-inhibitor functions upstream of C3 activation, it inhibits only for the classical and lectin pathways of complement activation.

## Methods

### Study Design and Animals

This study utilized three animal treatment groups: naïve (no CCI injury and no treatment), vehicle (CCI injury and i.v. PBS treatment), and CR2-Crry (CCI injury and i.v. CR2-Crry treatment at 16 mg/kg). Male C57BL/6J mice were purchased from Jackson at 8–10 weeks of age, acclimated to our animal facility for at least one week, and then surgeries performed at 10–12 weeks of age. Prior to surgeries, animals were randomly assigned to groups.

### Recombinant Proteins

Complement inhibitor CR2-Crry was expressed and purified in our lab as previously described (45). All proteins used in these experiments were subjected to quality control for complement inhibitory activity using a zymosan assay as previously described (45).

### Controlled Cortical Impact Model

The surgical model was performed as described previously (7, 18). Mice were anesthetized with an injection of ketamine (80–100 mg/kg, i.p.) and xylazine (10 mg/kg, i.p.). The surgical site was shaved and sterilized using three alternating treatments of Betadine and alcohol. Mice were head-fixed in a stereotactic frame (Kopf Instruments). A longitudinal midline scalp incision was performed, and a circular skull flap 4 mm in diameter was removed over the right cortex using a handheld drill (RWD Life Sciences). The impactor tip of the pneumatic impactor device (Infinite Horizon, Precision Scientific Inc.) was positioned over the surgical site (midway between lambda and bregma, centered 0.5 mm right of midline) and retracted. The impactor parameters were set to a tip size of 3 mm, depth of 2.5 mm, velocity of 5.25 m/s, and a dwell time of 100 ms. Mice received a single impact to the brain on the intact dura, then were removed from the device, given a surgical staple and returned to their home cage on a heat pad. Sham animals were not used because the craniotomy surgery can induce inflammation in the brain (46). Mice were monitored until awake and moving around the home cage. Animals were treated one hour after impact with an i.v. dose of 16 mg/kg CR2-Crry or with PBS vehicle. All animal experiments were performed after approval by the Institutional Animal Care and Use Committee (IACUC) at the Medical University of South Carolina.

### Optometry

Visual acuity was measured as previously described using the OptoMotry setup (Cerebral Mechanics, Lehtbridge, Alberta, Canada) (15). Briefly, mice were acclimated to the testing room and then placed on an elevated circular pedestal surrounded by four computer monitors. Visual recordings of mouse head responses to a rotating vertical grating were recorded using an overhead camera. Visual acuity results were based on a constant rotational speed of 12 degrees per second and 100% contrast using a staircase procedure. A mean screen luminance of 52 cd m^− 2^ was used for optomotor tests.

### Visual Cliff

The visual cliff was set up as described (47). Briefly, animals were placed on the edge of a checkered cliff, with alternating placement facing to the left or to the right. The surface (safe side or cliff side) that the animal placed its first step towards was recorded. A total of ten trials per side were performed per animal.

### Barnes Maze

Barnes maze was used to assess spatial learning and memory after TBI using a published protocol (48). Briefly, animals were trained to enter the escape hole for two trials per day for 5 days starting at day 21 after TBI. The escape hole was always in the same position relative to large fixed high-contrast visual cues. Videos were recorded and automatically analyzed using Noldus EthoVision XT.

### Optical Coherence Tomography

Optical coherence tomography measurements were performed using the Bioptigen spectral-domain optical coherence tomography system (Bioptigen, Durham, NC, USA) as described in (49). Mice were anesthetized with i.p. injections of ketamine and xylazine (as above), and eyes were dilated with phenylephrine and atropine. Sterile lubricant eye drops were applied often to keep the eyes moist. Circular volume scan images with diameter 1.6 mm were collected, consisting of 100 B scans. The thickness of each retinal layer was automatically calculated using Diver software.

### Tissue Processing and histological analyses

Animals were euthanized at either 3 days or 49 days after TBI surgery. Following euthanasia, mice were transcardially perfused with ice cold PBS followed by 4% paraformaldehyde dissolved in PBS, pH balanced to 6.9. Brains were extracted and placed in 4% paraformaldehyde solution overnight at 4°C. Brains were cryoprotected using 30% sucrose dissolved in 4% paraformaldehyde. Brains were frozen in optimal cutting temperature compound (Tissue-Tek) and cut into 40 μm sections using a cryostat (Leica).

### Immunofluorescence staining and imaging

Coronal brain sections containing the dorsal LGN (roughly section 76/132 from the Mouse Allen Brain Atlas) were selected and stained by a standard immunofluorescent staining protocol as described previously (18). High-resolution imaging was performed using a Zeiss LSM 880 confocal microscope (Zeiss, Carl Zeiss Microscopy, LLC, White Plains, NY, USA) at 63 x with oil-media overlay and using the Z-stacking feature. The dLGN was identified by anatomic reference to a standard coronal section. Imaris Microscopy Image Analysis Software was used for analyses as described in (50). For synaptic or C3 colocalization, surfaces were drawn corresponding to C3, VGLUT2, or PSD95, and the fraction of one object within 0.2 microns (the width of the synaptic cleft) of another was determined and quantified as physiologic association. For microglial internalization, surfaces were determined based on microglial stain, and full microglia were identified based on staining pattern. New surfaces were created using the VGLUT2 or C3 stain within the boundaries of the microglial surface, which was used to determine the volume to volume ratio of internalized material to microglia. For morphological analysis, background was subtracted and 3D renderings of microglial filaments were created and manually checked using the “FilamentTracer” tool. Microglia filament lengths, total volume, and number of processes were automatically quantified. For retinal stains, 20 x images of retinas were acquired using a Keyence BZ-X710 microscope. C3 intensity and inner and full retina area were quantified using NIH Image J.Primary antibodies used for staining were: anti-C3 (Abcam, Cat. #: ab11862, 1:200), anti-IBA1 (Invitrogen, Cat. #: PA5–21274, 1:100), anti-VGLUT2 (Abcam, Cat. #: ab216463, 1:200), anti-PSD95 (Abcam, Cat. #: ab18258, 1:200). Secondary antibodies used were all donkey and were anti-rabbit Alexa Fluor 488 (Invitrogen, Cat. #: A-21206, 1:200), anti-rat Alexa Fluor 555 (Abcam, Cat. #: ab150154, 1:200), and anti-goat Alexa Fluor 647 (Invitrogen, Cat. #: A32849, 1:200).

### Statistical Analysis

Statistical analysis and data representation were performed using GraphPad Prism 8.0 (GraphPad Software, San Diego, CA, USA). Details on statistical tests used are included in the figure legends. All data in the manuscript are represented as mean ± s.e.m. and p values < 0.05 were considered significant. Sample size estimation was done based on previous work from the lab with an acceptable power range of 85–90%.

## Data Availability

All data generated and analyzed to support the findings of this study are included within the article. Additional data sets are available upon request.
